# Eating behaviors, attitudes, and beliefs that contribute to overweight and obesity among women in Lilongwe City, Malawi: a qualitative study

**DOI:** 10.1186/s12905-022-01811-0

**Published:** 2022-06-09

**Authors:** Myness Kasanda Ndambo, Alinane Linda Nyondo-Mipando, Chrissie Thakwalakwa

**Affiliations:** 1 School of Global and Public Health, Kamuzu University of Health Sciences, Chichiri, Private Bag 360, Blantyre 3, Malawi; 2 Centre for Social Research, Chancellor College, University of Malawi, Zomba, Malawi

**Keywords:** Obesity, Overweight, Eating behaviors, Attitudes, Beliefs, Developing countries, Malawi

## Abstract

**Background:**

Obesity is increasingly a public health concern in low- and middle-income countries, including Malawi where 36% of women have body mass index in overweight/obese categories in urban areas. Eating behaviors, attitudes, and beliefs are associated with body size, but have not been studied in-depth in sub-Saharan African countries. This study therefore, explored eating behaviors, attitudes, and beliefs of women in Lilongwe, Malawi.

**Methods:**

This was a descriptive ancillary qualitative study utilising in-depth interviews with 27 women (13 in normal weight range and 14 in overweight/obesity ranges) puporsively selected in Lilongwe City, Malawi from October to November 2017. The concept of data saturation guided data collection, and it was reached with the 27 interviewed participants when there was no new information coming from the participants. All interviews were conducted in the local language, transcribed verbatim, and translated into English. The transcripts were analysed manually using thematic content analysis.

**Results:**

Majority of participants perceived overweight as an indication of good health such that with food affordability, women deliberately gain weight to demonstrate their good health. Most normal weight respondents said they ate less food than they wanted to because of financial constraints. Most women in overweight/obese ranges in our sample reported that they eat large portions and eat frequently due to the desire to portray a good image of their marital life since there is a societal expectation that when a woman is married, her weight should increase to show that the marriage is successful. The perceived contributors to weight gain include eating behaviors, feelings about weight gain, and gender roles and social expectations to gain weight.

**Conclusion:**

Beliefs and attitudes related to eating behaviors may have contributed to women being in overweight range and should be considered in designing obesity prevention interventions targeting women in Malawi.

**Supplementary Information:**

The online version contains supplementary material available at 10.1186/s12905-022-01811-0.

## Background

Overweight and obesity rates are increasing globally, especially among women in urban settings [1–4]. In 2016, 1.9 billion (39%) adults aged 18 years and above (39% of men and 40% of women) were in overweight categories and 13% of the same adult population (11% of men and 15% of women) were in obese categories [5]. Though previously considered as a problem of high-income countries, obesity and overweight rates are rising tremendously in low and middle-income countries [5]. Urbanization and globalization have contributed to the increase in overweight and obesity rates through increased access to energy-dense foods, including high-calorie fast foods, packaged and processed foods, and sugar-sweetened beverages, and decreased physical activity secondary to advancements in technology [6–9].

Eating behavior, the patterns of an individual’s food and beverage intake practises indicated by food choices, meal frequency, and portion size [10], is an important aspect of the rising prevalence of overweight and obesity. Among healthy, nonsmoking women followed over 20 years, disinhibited eating was consistently associated with high body mass index (BMI) and susceptibility to overeating in response to everyday cues within one’s environment was the strongest correlate of weight gain [11]. Individuals in low-income families tend to overeat as a response to chronic or periodic food insecurity, and rely on inexpensive sources of calories, especially starchy staples [12]. Overeating; alcohol use; increased consumption of meat, dairy, and sugary products; and decreased physical activity contribute to overweight and obesity rates among more affluent families in Sub Saharan Africa (SSA) [12–14]. A negative perception of thin body size is also a risk factor for overweight and obesity [7, 15, 16]. In SSA, larger body sizes are associated with beauty, prosperity, health, and prestige, while thinness is perceived as a sign of ill health or poverty [17–19]. Women may therefore eat surplus amounts of food to gain more weight and look healthy.

In Malawi, literature indicates that women dwelling in urban settings are prone to overweight and obesity as opposed to their rural dwelling counterparts [4]. This is in line with reports by the Malawi Demographic Health Survey 2015/16, which indicate rising rates of overweight in urban women (36%) than in rural women (17%) [20]. The proportion of Malawian women who are in overweight or obese ranges has increased steadily, from 10% in 1992 to 21% in 2015–2016 [20, 21] and Malawian women have a preference for larger body sizes, [20, 22–24]. These increasing rates of overweight and obesity are likely to lead to high incidence of cardiovascular diseases and diabetes if measures are not taken to curb the problem, especially in urban women [25–27]. The formulation and deployment of such measures is dependent on availability of rich knowledge and information on overweight and obesity. However, there is a dearth of information on women’s eating behavior linked to their attitudes and beliefs towards overweight and obesity in urban Malawi. To address this gap, this paper explored eating behaviors, attitudes, and beliefs of women in Lilongwe, Malawi.

## Methods

This was a descriptive ancillary qualitative study utilising individual in-depth interviews (IDIs) with women in normal weight and overweight/obese ranges. The in-depth interviews provided an opportunity for a detailed investigation of each individual’s perspective [28]. This was a sub study of a mixed methods study of drivers of food choice in Malawi which purposefully enrolled women in normal weight, overweight, and obese categories to identify cognitive, cultural, economic, gender, and seasonal predictors of dietary intake and food choice [24, 29, 30]. This sub-study focused on women’s perceptions of the factors that influence their eating behaviors. Eating behavior plays an important role in healthy eating [11, 31, 32].

### Study sample

The study purposively selected 32 women, in Area 18 and Kawale townships in Lilongwe City, from a list of women who participated in a larger study on drivers of food choice in Malawi. Purposive sampling is the deliberate selection of the informants based on the qualities they possess [33]. An equal number of women in normal weight (n = 16) and overweight/obese (n = 16) categories were selected based on their BMI, using standard cut-offs [normal weight (BMI 18.5–24.9 kg/m^2^) and overweight (25.0–29.9 kg/m^2^) or obese (BMI > 29.9 kg/m^2^)]. Within each weight category, the sample was equally divided between the two study sites (Area 18 and Kawale). The study included all women within normal weight, overweight and obese categories with ages ranging from 19 to 39 and excluded all pregnant women, due to expected increase in BMI among this group. The sample size was preselected because it was more than the minimum usually needed to achieve saturation, the point in qualitative research at which the data collection process no longer offers any new data [34, 35]. Previous research has shown that a minimum of 6–12 in-depth interviews is needed to reach saturation [36]. Saturation was reached with the 27 interviewed women, so there was no need to seek additional respondents.

### Data collection

Data was collected by one of the researchers (MKN) and one trained research assistant using an in-depth interview guide (Additional file 1: Appendix). The guide was developed for the purpose of this study, pretested with 10 women in Area 25 Lilongwe to ascertain validity and consistency with the objectives of the study, and amendments were made as necessary before the actual data collection. The facilitator explained the study to participants and obtained signed written informed consent. The study was conducted by the Declaration of Helsinki guidelines and regulations [37]. All interviews were held only once, in participants’ homes, were face-to-face, and were digitally audio recorded. Interviews were conducted in Chichewa, the local language. The interviewers captured field notes to complement audio recordings during analysis. These field notes acted as cues for reflection and supported initial coding [38]. To enhance credibility of our findings, the researcher summarized key findings to participants at the end of each interview to verify them [39]. MKN and CT held multiple discussions over the data and interpretations to achieve consensus and dependability [39]. The interviews ranged from 20 to 35 minutes long. The concept of data saturation guided data collection, and it was reached with the 27 interviewed participants when there was no new information coming from the participants [35].

### Data analysis

Data was analysed manually through thematic content analysis [40–42]. MKN listened to the audio recordings multiple times and read transcripts repeatedly to become familiar with the data whilst paying attention to significant aspects for further discussion. During the reading phase, discussions were held between MKN and CT to clarify the data to ensure that there was no misrepresentation of the data. After multiple discussions, a coding framework was developed by MKN from two sampled transcripts, double checked by CT and then the rest of the transcripts were deductively coded by MKN. All similar codes were grouped under an overarching theme through an iterative process to attain the best fit for the codes. Unsatisfactory codes were dropped during refinement, following discussions between MKN and CT. Similar codes were then grouped. Finally, emerging relationships were carefully examined and categorized into themes which were addressing the study objectives [40]. The themes were reviewed in line with their quotes to avoid losing meaning.

## Results

The total number of interviewed participants was 27. The targeted sample size (32) was not attained because two women in Kawale and three women in Area 18 had moved to other locations. Participant’s ages ranged from 19 to 39 years with the interquartile range being (23,32). Twenty-two percent of the women attended tertiary education, 44% secondary, 30% primary, and 4% never attended school. Seventy-four percent of the women were married. Based on occupation, 41% were housewives, 44% were doing business and they spent more time at home, and 15% were employed. Based on BMI, 48% of the women had a normal weight, 26% were overweight and 26% had obesity.

### Women’s perceptions of the factors that contribute to their body size

Women’s perceptions of the factors that contribute to their body size revolve around eating behaviors, feelings about weight gain, and gender roles and social expectations as presented below:

### Eating behaviors among women in normal weight category and overweight/obese ranges

One third of women in normal-weight category (educated) were dieting to keep their bodies fit whilst two thirds of them (not educated) said they ate less food than they wanted to because of poverty. Surprisingly, no woman in overweight or obese categories indicated dieting or practicing any method to reduce weight. Some normal weight respondents gave the following reasons for their eating practice:

As for me, I diet most of the times because I want to maintain my body weight and keep fit. **Normal weight woman, 36years, Area 18.**

I restrict my eating because of financial problems. Sometimes I eat two times a day while sometimes I eat once a day because that is what I can manage. That is why I am falling on a normal weight category, however, if I had enough, I could not be like this. **Normal weight woman, 25years, Area 18.**

Women in overweight and obese categories were not open enough to immediately describe their eating behaviors. However, probing revealed that these respondents tend to eat large portions and they eat frequently. In addition, no woman in this category complained about financial related problems as regards food availability. Here is an example of eating behaviors from an obese respondent:

Aaah, me I do not play with food. When it comes to eating, I eat. I make sure I eat that which I want… **Obese woman, 23 years, Kawale.**

### Feelings about weight gain that affect women’s attitude towards gaining weight

Women discussed some affective attitudes (feelings) that encouraged them to gain weight. Women related overweight to being health and looking beautiful which eventually encouraged them to gain weight. However, it was interesting to note that both women in normal weight categories and overweight/obese ranges viewed overweight from the same angle, that it signifies beauty. Apart from the one third (dieting) women in normal weight category, all women worked towards improving their weight. Therefore, women reported eating frequently in order to look beatiful.

…even though I am falling under normal weight range, it is against my wish, when you are fat you look nice and beautiful. I try to eat frequently (mwakathithi) but I am not getting there, I think I was just meant to be like this. **Normal weight woman, 30years, Kawale.**

Women cited family planning as a major contributor to their weight gain hence take it with an objective of gaining weight. This theme recurred throughout the interviews. However, the same respondents also reported eating large portions. Below is an example of respondents’ views towards the influence of family planning methods on body size:

If you do not want us to gain more weight, do something on family planning methods...we are gaining too much weight because of the same. Some women even take these methods not necessarily to prevent pregnancies and STIs but also to gain weight. When I started taking birth control pills, I gained weight immediately. **Obese woman, 32 years, Area 18.**

However, some women linked their weight gain to their occupation. Women who were housewives attributed their weight gain to idleness. Without engagement in any economic activity that would keep them busy, at-home women resort to food and eating to pass their time. For example:

Maybe if I start a business I can keep myself busy and burn some fats. Being at home all the time makes me idle. All I do is eat. **Overweight woman, 34 years, Area 18**.

Women who had jobs or businesses attributed their weight gain to unhealthy eating behaviors at their workplaces, including skipping meals and eating food prepared in restaurants. One respondent said:

I gain more weight because of my unhealthy eating behaviors when I am at work. Sometimes I skip meals because I am busy and then take a bigger portion afterwards. I also eat fatty foods from restaurants most of the time. **Obese woman, 39 years, Area 18.**

### Gender roles and social expectations

Women reported factors such as childbearing, responsibility for cooking food for the family, women’s decision-making about food preparation, and the social expectation for women to gain weight after marriage as contributors to overweight/obesity.

#### Childbearing as a contributor to weight gain

Women believe that giving birth creates a large hole in the stomach that needs to be filled with food. This belief leads them to eating large portions (surplus amounts) of food to fill the hole. They also said that their appetite increases when they are breastfeeding, causing them to eat more and gain weight. Respondents explained these beliefs as follows:

When I delivered my first baby, my mother-in-law told me that I should eat a bigger portion since the baby has left space in my stomach and this space needs to be filled up. Hence, I increased in weight. **Obese woman, 24 years, Kawale.**

My weight changed with childbearing. Before I started bearing children, I was underweight, in fact, but after I delivered my first child my appetite increased and I automatically started gaining weight. **Normal weight woman, 26 years, Kawale.**

#### Responsibility for cooking food for the family

Women reported that they usually prepare food for their families and they eat frequently because they are exposed to food as they prepare it. Women said it is not easy to be on diet while cooking, adding that cooking is a temptation to women who are at home most of the time. One respondent explained:

Sometimes I feel like dieting so that I may lose some weight. However, being in the kitchen cooking nice food for my family puts me into temptations. I believe in testing food while cooking hence I find myself eating when I did not plan to eat. **Obese woman, 30 years, Area 18.**

#### Women’s decision-making about food preparation

All women said that when they were young and staying with their parents or guardians, they were restricted to eating what had been prepared at home. Now that they are managers in their own homes, they can act on their own (agency). In other words, they can eat what they want at any time as long as they can afford such foods. One respondent explained:

When I was young, I was restricted to eat what my mum prepared. I did not dare to eat the food of my choice. Now that I have my own home, I am free to eat whatever I want…because it is my money. **Overweight woman, 22 years, Kawale.**

#### Social expectations for women to gain weight after marriage

All women expressed a belief that when they get married, they are expected to gain weight as an indication that they are happily married and stress-free as explained below:

Yes, when you are married, people expect you to look more improved, otherwise they can conclude that things are not going on well in your family, be it financially or health-wise. Therefore, we eat to make sure that people should see a difference. **Normal weight woman, 35 years, Area 18.**

When you are thin, people relate that to your marriage. They conclude that your marriage is not going on well, such that you do not have peace in your family. So, because of that, I try to eat frequently so that I can look good. **Obese woman, 46 years, area 18.**

## Discussion

Our study reveal that there are a number of eating behaviors, attitudes, and beliefs that contribute to weight gain among women in urban areas. These include eating behaviors among women in normal weight range and overweight/obese categories, feelings about weight gain that affect women’s attitude towards gaining weight, and gender roles and social expectations. The study noted no difference in beliefs in the context of overweight among women in normal weight range and those in overweight/obese categories. However, attitude differences existed as one third of women in normal weight category reported dieting and these attained secondary and tertiary education while two thirds (primary school drop outs) were falling in the normal weight category because of financial constraints.

Our study has shown that women in overweight and obese categories eat larger portions and they eat frequently. This is shown where such women would come out openly to say they do not play with food. It is also worth noting that in tandem with the increasing prevalence of overweight and obesity, there is a rise in food portion size [43, 44]. The problem is that people are facing difficulties in estimating [45] the amounts of food and there is inadequate knowledge of reference portion sizes [46] as such, individuals are at risk of consuming large portions (surplus amounts) when food is in abundance. This might imply that socioeconomic status increases the prevalence of overweight and obesity in urban women. Again, 57% (8/14) of women in overweight and obese categories in this study had a secondary or tertiary level of education. This may also imply that education is related to increased socioeconomic status such that the higher the educational level the higher the food availability. The Malawi Demographic and Health Survey 2015–2016, showed that overweight and obesity increases with education and wealth [20]. Similar findings were noted in studies conducted in Zambia and Kenya where higher educational attainment was positively associated with women being overweight or obese [14, 47]. Further investigations are needed to identify gaps in education that could be used to empower the educated with obesity prevention measures.

Our study has shown that there are feelings towards overweight that affect women’s attitude. Women in this study perceive overweight as a sign of health and well being as well as beauty. This feeling prompts women to eat frequently and gain weight. In this study, family planning is perceived as a contributor to weight gain as some women opt for combined oral contraceptive (COC) pills not just to prevent pregnancy but also to gain weight. A similar attitude was reported in Britain where a decrease in uptake of COC pills among adolescents was noticed for fear of gaining weight [48]. However, a comprehensive literature search did not find evidence for the purported weight gain with use of low dose COCs [48]. There is need therefore to dispute such misconceptions among women since they might practice poor eating behavior whilst on COC pills and associate weight gain to the same.

Notably, this study has also shown that both at home and away from home occupations contribute to weight gain. Women working at home are exposed to food and they tend to eat frequently. Women working away from home are also exposed to unhealthy foods in restaurants. From a different perspective, a similar study examined the influence of occupation on eight hours of sitting daily and weight change in employed women and reported a profound influence of weight gain over time [49]. Further investigations would be required to find best interventions that can prevent overweight and obesity in both women working at home and away from home.

In this study, gender roles is used to refer to reproductive work done by women especially child bearing, women’s cooking responsibility for their families, and women’s decision making about food preparation. Women in this study believe that childbearing contribute to increased BMI. Women believe that after delivery they should eat large portions to cover up the hole or space left behind by pregnancy as well as to have enough nutrients for the baby. This may result in excess food than the body’s energy requirements. Literature shows that excess food intake occurs when energy intake exceeds the body's physiological needs, which eventually leads to a positive imbalance between energy intake and energy expenditure hence leading to overweight [50, 51]. These findings are similar to a study where African-American women 21–69 years old were enrolled in a long-term health study whose results indicate that childbearing among African-American women increases weight gain [52]. An increase in BMI is noted in many women during postpartum with a very minimal decrease afterwards.

The study findings have shown that women’s roles in a family influence weight gain. As the person in the family responsible for cooking, they are also exposed to food as they prepare it, which encourages them to eat more. Women’s decision making about food preparations in the house also expose them to overeating since they are at liberty to eat anything they want as opposed to the time when they were kept under parental control. This might mean that cooking role and food preparation decision making increases their agency and puts them in control there by increasing food desirability more especially when food is available.These results also mirror Thakwalakwa et al. who reported food affordability and desirability as the most common predictors for overweight and obesity [29].

Social expectation in this study refers to behavioral expectations of the society on someone. Expectations influence social behavior and are capable of determining the behaviour of an individual, community and a large mass of people [53]. For instance, this study has shown that there are social expectations that women should gain weight after getting married to show that they are doing well. Such expectations build up a desire in women to prove their marital success. As a result, women overeat depending on food availability to portray a good image of their marital life. This is consistent with findings from a previous study showing that marital status is an important determinant of weight gain [54] and obesity was earlier reported as highest in middle-aged housewives with middle and high social-economic status [55]. However, some educated women are able to operate outside this belief by maintaining normal weight through dieting. This may imply that education could be successfully used as a channel to eliminate harmful beliefs in the communities in the course of fighting obesity.

### Limitations

This study was limited to particular participants as it was embedded in another study hence there was no room to conduct purposive sampling in this ancillary study. Again, data collected in this study cannot be generalised for the country due to its qualitative nature. Even though some study participants reported eating large portions, this study did not systematically manipulate portion sizes and their effects hence this data do not infer causality. However, the results of this study are a stepping stone for interventions in decreasing the prevalence of overweight and obesity in the country including public acceptance of portion size interventions and portion size policies.

## Conclusion

The findings of this study suggest that overweight and obesity in urban women are linked to unhealthy eating behaviors, attitudes, beliefs, and exposure to food. Most women in overweight range in our sample eat large portions and eat frequently. Women might not be aware of the health implications of obesity, hence the preference to gaining weight because they want to show that they are doing well in life. The results of this study have public health implications that are crucial for policy implementers and program planners in the field of nutrition.

This study therefore, recommends encouragement of health promotion initiatives as preventive measures to overweight/obesity. As the Malawi Ministry of Health is implementing integration of health services, it is imperative to include a detailed package on obesity prevention through health talks during antenatal visits as well as under five clinics with more emphasis on behavior change in diet and awareness of potion size references. Finally, research is needed to determine the effectiveness of different strategies for overweight and obesity prevention.

## Supplementary Information

**Additional file 1**. In-depth Interview guide.

## Data Availability

The datasets generated and/or analysed during the current study are not publicly available as the research team is currently analysing the data to answer other research questions, but are available from the corresponding author on reasonable request.

## Abbreviations

BMI: Body mass index
SSA: Sub Saharan Africa
IDIs: In-depth interviews
MDHS: Malawi Demographic Health Survey
COC: Combined oral contraceptive
LMIC: Low and middle income countries
