# Supplementary material for: Eating behaviors, attitudes, and beliefs that contribute to overweight and obesity among women in Lilongwe City, Malawi: a qualitative study
Source: BMC Womens Health. 2022 Jun 9;22:216. doi: 10.1186/s12905-022-01811-0 (PMC9185864; doi:10.1186/s12905-022-01811-0)
Supplement: Supplementary file 1 — Additional file 1. In-depth Interview guide. [file 12905_2022_1811_MOESM1_ESM.docx]

**Eating Behaviors, Attitudes, and Beliefs that Contribute to Overweight among Women in Lilongwe City, Malawi: A Qualitative Study**

**In-depth Interview guide**

**SECTION A: DEMOGRAPHIC INFORMATION (to be obtained from DFC data)**

Participant DFC ID: ------------ Date: -----------------Name of Interviewer: -------------Weight: ---------------Height: -------------BMI: --------Category: --------------------------Age: -----------Marital status: ----------Occupation: ------------Education: --------Area: -------

**SECTION B: INTRODUCTORY QUESTION**

1. How did you hear about our meeting/ interviews today?
2. Do you think overweight or obesity is an issue of concern in this area?

**SECTION C: TRANSITION QUESTIONS**

- 1. What kind of foods do you like?
  2. Why do you like these kind of foods?
  3. Where do you eat these kinds of foods?
  4. How many times do you eat per day?

**SECTION D: KEY QUESTIONS**

- 1. Please describe your eating behaviours/ practices.
  2. What factors influence the way you eat? Which of the factors you mentioned has the biggest influence on your eating habits?
  3. How have your eating behaviours changed since you got married?
  4. How have your eating behaviours changed since you started bearing children? ***Probe:*** Changes in types of food consumed, changes in frequency of eating
  5. (If eating behaviours changed, the interviewer should ask this question)What would you say caused these changes?
  6. When you think of healthy eating behaviours, what comes to your mind?
  7. How do you think your eating behaviours compare with the healthy eating behaviours you just described?
  8. (To those who do not follow) What do you think you could do to change your eating behaviours so they are healthier?

**SECTION E: ENDING QUESTIONS**

- 1. If the Government of Malawi wants to help women make healthier eating choices to avoid being overweight, can you suggest some ideas/ advice on how to promote healthy eating behaviours in women?
  2. Do you have any other suggestions and additions?

**Thank you for participating in this study**!
